# Acute Limb Ischemia in a Patient With COVID-19 Pneumonia

**DOI:** 10.7759/cureus.18574

**Published:** 2021-10-07

**Authors:** Shahbaz Ali Nasir, Anum Arif, Mubasshar Shahid, Yashfeen Ahmed, Bismah Riaz, Nawabzada Zeerak Farhat Sherwani

**Affiliations:** 1 Internal Medicine, Combined Military Hospital, Lahore, PAK; 2 Vascular Surgery, Combined Military Hospital (CMH) Lahore Medical College, Lahore, PAK; 3 Internal Medicine, Combined Military Hospital (CMH) Lahore Medical College and Institute of Dentistry, Lahore, PAK; 4 Internal Medicine, Combined Military Hospital (CMH) Lahore Medical and Dental College, Lahore, PAK

**Keywords:** comorbidities, limb ischemia, coronavirus, pneumonia, thromboembolism

## Abstract

COVID-19, which is caused by the novel severe acute respiratory syndrome coronavirus (SARS-CoV-2), is known to cause a myriad of complications along with the typical lower respiratory tract involvement. One of the emerging complications is a hypercoagulable state leading to venous or arterial thromboembolism. These complications are more common in those presenting with a severe disease with significantly elevated inflammatory markers. Although co-morbid illnesses play a role in worsening such complications, yet they are not the main determinants as these complications also occur in those without any co-morbid illness. Here, we report a case of a 64-year-old male with severe COVID-19 pneumonia presenting with acute limb ischemia with a non-salvageable limb who required subsequent amputation of the affected limb.

## Introduction

Since December 2019, when the first case of coronavirus was reported, there has been a constant evolution in the clinical presentation of the disease from simple viral-like illness, i.e., fever, sore throat, myalgia to acute respiratory distress syndrome (ARDS), multi-organ failure, and thrombotic events [1-3]. One of the proposed mechanisms contributing to the disease’s multi-organ involvement is endothelial dysfunction leading to increased levels of pro-inflammatory cytokines such as interleukin-6 along with elevated levels of acute-phase reactants such as D-dimer [4]. There have been a few studies done that have noticed an increased incidence of thrombotic complications involving both the arterial and venous systems [5]. We would like to discuss here a case that presented with acute lower limb ischemia due to COVID-19.

## Case presentation

A 64-year-old male with a past medical history of type 2 diabetes mellitus and essential hypertension presented in the emergency department with a history of intermittent fever of 102 degrees Fahrenheit for the last five days along with numbness and discoloration in his right leg for the last three days and shortness of breath. On examination, his Glasgow coma scale (GCS) score was 15/15 and his mental status was normal on gross neurological examination. His oxygen saturation (SpO_2_) at room air was 85% (indicative of severe disease) and 94% SpO_2_ at 15 L oxygen via a non-breather mask (NRM). 

**Figure 1:**
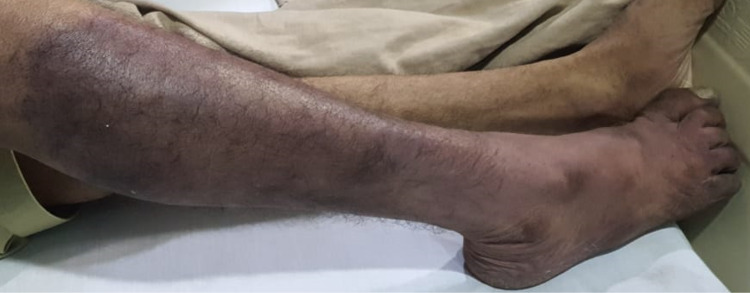
Blackish discoloration of his right leg with a line of demarcation just below the knee joint

On physical examination, his right leg had a blackish discoloration (Figure 1).

**Figure 2:**
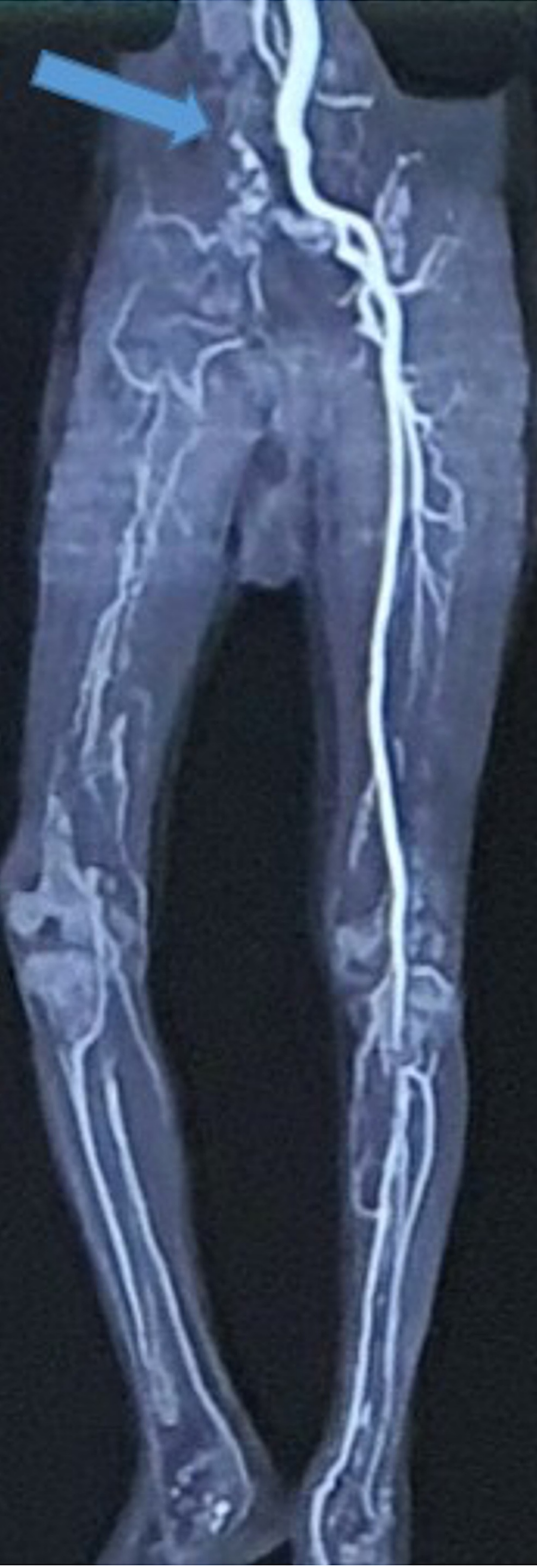
Computerized tomography angiography (CTA) (coronal view): The blue arrow indicates sudden abruption of contrast from right common femoral artery (CFA) and no contrast opacification in the popliteal artery (PA), anterior tibial artery (ATA), posterior tibial artery (PTA), and dorsalis pedis artery (DPA)

It was cold, clammy, with an absent femoral pulse. Arterial Doppler ultrasound of the right lower limb showed no flow in the femoral, popliteal, anterior tibial, posterior tibial, and dorsalis pedis arteries although no plaque or echogenic thrombus was seen. A CT angiogram of the lower limb was performed, which showed no contrast opacification beyond the right common femoral artery (Figure 2).

A Vascular surgeon was consulted and a diagnosis of acute limb ischemia was made. It was classified as Rutherford stage III, which was three days old. The limb was declared as non-salvageable. Keeping in view the clinical picture and CT angiogram findings, the Vascular surgeon advised an above-knee amputation of the affected limb.

**Figure 3:**
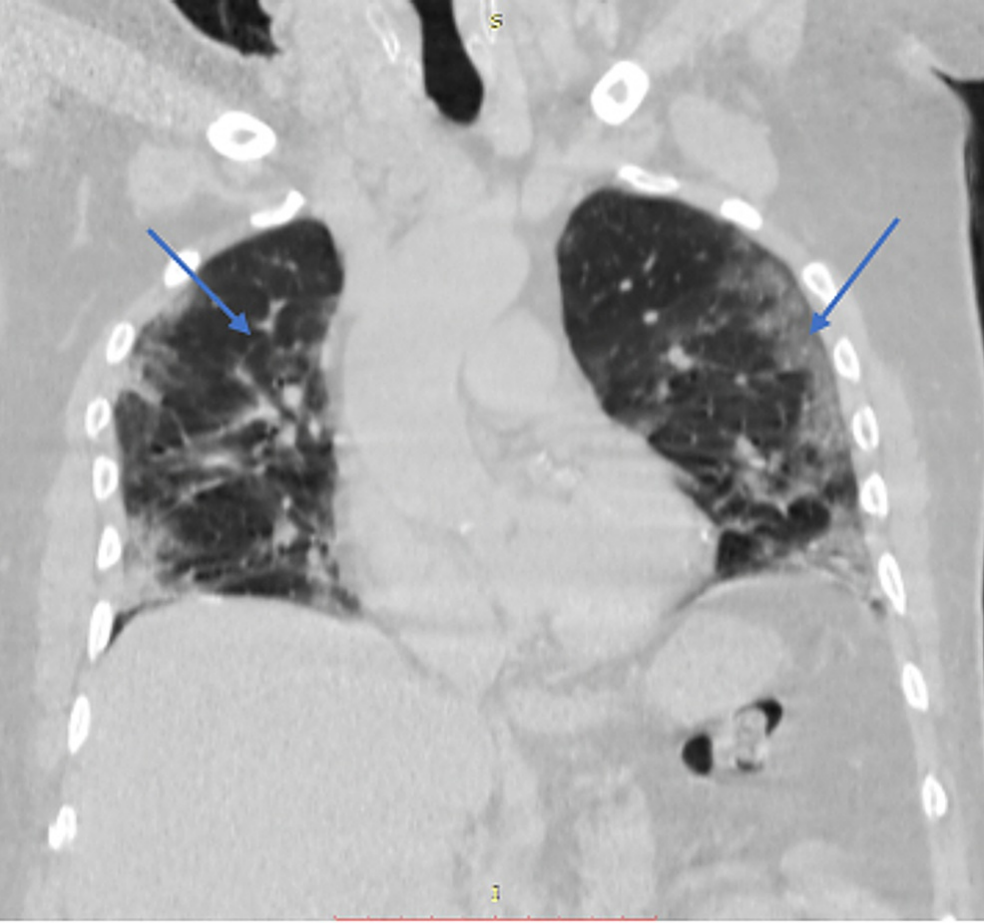
High-resolution CT (HRCT) scan of the chest with a coronal view of the patient with ground glass opacities bilaterally as shown by arrows with a CT severity score of 31/40

 The patient's real-time SARS-CoV-2 polymerase chain reaction (PCR) test was positive. His arterial blood gases (ABGs) showed metabolic acidosis with respiratory compensation with a pH of 7.4, bicarbonate (HCO^3-^) of 17.9, and pCO_2_ of 25.9. His high-resolution computerized tomography (HRCT) of the chest gave a severity score of 31/40 for COVID-19 pneumonia (Figure 3), indicative of severe disease.

His serum ferritin levels were 1,279 ng/mL (N=15-200 ng/mL), serum D-dimer 1,650 ng/mL (N≤250 ng/mL), serum LDH 861 U/L (N=135-225 U/L), serum CK 4,252 U/L (N= up to 192 U/L), INR=1, IL-6=14.8 pg/mL (N≤7.0 pg/mL). CBC showed WBC count of 29.0 x 10^9^/L with neutrophil-lymphocyte ratio (NLR) of 22.25 (N=1-3). His HbA1c was 12.4 (N=4.5%-6.5%).

The patient's above-knee amputation was done under spinal anesthesia, and he was subsequently moved to the COVID-19 Intensive Care Unit, where he was started on injection meronem, dexamethasone, and remdesevir, as per local protocol. He was also given intravenous fluids, pain medication, and insulin according to a sliding scale. After 12 hours of surgery, he was given therapeutic anticoagulation with enoxaparin.

After eight days, the patient's clinical state improved, with oxygen saturation increasing to 94% on room air. His inflammatory markers were within the normal range following 12 days of sickness. On day 15, he was discharged with instructions for chest physiotherapy and incentive spirometry, as well as wound care and pulmonology, vascular surgery, and rehabilitation clinic follow-up.

## Discussion

Acute limb ischemia is defined as a sudden decrease in limb perfusion that threatens the viability of the affected limb [6]. The incidence of this condition is approximately 1.5 cases per 10,000 persons per year. The clinical presentation is considered to be acute if it occurs within two weeks after symptom onset [7].

Acute limb ischemia resulting from COVID-19 is a rare occurrence but is emerging nowadays, hence there is very limited data on the disease epidemiology and management. One study done in a New York City hospital in the U.S.A. identified 49 patients out of 12,630 hospitalized with COVID-19 that developed arterial thromboembolism of which seven (14%) had upper limb ischemia and 35 (71%) had lower limb ischemia [8]. By comparison, the rate of ALI in the general population before the COVID-19 pandemic is approximately 10 to 15 per 100,000 per year and includes embolic, thrombotic, and traumatic etiologies [9]. In our patient, on detailed assessment, no other thrombotic or embolic cause was found to determine the possible cause of ALI other than COVID-19.

Bellosta et al. conducted a cohort observational study in Italy in 2020 showed a significantly greater number of patients with ALI as compared to the same period in 2019 before the COVID-19 pandemic. They also projected a fivefold increase in vascular intervention when compared with 2019 [10]. Roquetaillade et al. reported in their study conducted in France that 20 out of 209 patients developed arterial thromboembolic events. At the time of the incidents, 50% of them were undergoing thromboprophylaxis and two out of 20 people developed acute limb ischemia [11]. The literature search shows scanty evidence of any correlation between optimal timings and dose of anticoagulation in COVID-19. This is still grey area as COVID-19 has a diverse spectrum and guidelines are in infancy.

A study was conducted in August 2020 in four New York City hospitals to calculate the incidence and risk factors for venous and arterial thrombosis in hospitalized covid-19 patients. Among the total of 3,334 hospitalized COVID-19 patients, the median age was 64 years, 61.4% of these hospitalized COVID-19 patients were males and 39.6% were females. The author of the same study concluded that age, male gender, prior history of coronary artery disease, and elevated D-dimers at hospital presentation significantly raised the risk of thrombotic events in COVID-19 patients [12].

Several articles published recently showed the occurrence of arterial thrombotic events in COVID-19 positive patients with no previous history of peripheral arterial disease [13,14]. This is because Viral infection-induced inflammation has been recognized as a crucial component in the establishment of a pro-coagulant state. Tissue factor-dependent activation of the coagulation cascade as a result of endothelial cell damage and dysfunction increased levels of von Willebrand factor, and activation of pro-inflammatory cytokines are all variables that contribute to this occurrence [15]. Similarly, a study conducted in Kuwait presents five cases of COVID-19 positive patients with no significant comorbidities who developed first-time episodes of either acute limb ischemia or bowel ischemia. Examples such as this should alert clinicians about the possibility of acute limb ischemia in patients without the typical comorbidities predisposing them to acute limb ischemia [16].

A study conducted in Portugal showed the impact of COVID-19 on vascular intervention in the emergency department. According to the above-mentioned study, there was a rise in the number of urgent vascular interventions in the COVID-19 pandemic when compared to the preceding 10 years [17]. This can be explained by the fact that ALI needs a high index of suspicion but due to the fear of COVID-19 and the isolation of the patient, this is usually overlooked by the physician treating COVID-19. One other reason may be that patients are afraid to seek medical care until disease severity became difficult to manage medically.

Since 2020, the COVID-19 symptoms have been developing at a rapid pace. Initially, the focus was solely on respiratory symptoms, but recently, other significant symptoms such as covid-induced myocarditis, arthritis, liver impairment, encephalitis, and others have emerged. Acute limb ischemia is a difficult condition to treat since there is a time limit after which the limb cannot be salvaged. Since Pakistan is a developing country, there is a scarcity of vascular surgeons, and because everyone is focused on the respiratory aspect of the patient, these concerns may be missed by first-hand physicians. We wish to share our experience and add to the research on how a case of COVID-19-induced acute limb ischemia is managed in a developing nation like ours by reporting this.

## Conclusions

All COVID-19 patients including those with atypical presentation should be offered all baseline investigations including screening panels for thromboembolic diseases like serum D-dimers, serum LDH, serum ferritin, PT/APTT/INR irrespective of known co-morbidities. We further recommend that prophylactic anticoagulation be administered to all COVID-19 patients who require hospitalization, as well as to high-risk patients with previous venous thromboembolism (VTE), recent surgery or trauma, immobilization, or obesity as outpatient treatment.
